# The relevance of words and the language/communication divide

**DOI:** 10.3389/fpsyg.2023.1187343

**Published:** 2023-07-28

**Authors:** Robyn Carston

**Affiliations:** Linguistics, University College London, London, United Kingdom

**Keywords:** lexical pragmatics, relevance theory, metonymy, root-based syntax, non-compositional meaning, communicational lexicon, form/meaning divide, decoded linguistic meaning

## Abstract

First, the wide applicability of the relevance-theoretic pragmatic account of how new (*ad hoc*) senses of words and new (*ad hoc*) words arise spontaneously in communication/comprehension is demonstrated. The lexical pragmatic processes of meaning modulation and metonymy are shown to apply equally to simple words, noun to verb ‘conversions’, and morphologically complex cases with non-compositional (atomic) meanings. Second, this pragmatic account is situated within a specific view of the cognitive architecture of language and communication, with the formal side of language, its recursive combinatorial system, argued to have different developmental, evolutionary and cognitive characteristics from the meaning side of language, which is essentially pragmatic/communicative. Words straddle the form/meaning (syntax/pragmatics) divide: on the one hand, they are phrasal structures, consisting of a root and variable numbers of functors, with no privileged status in the syntax; on the other hand, they are salient to language users as basic units of communication and are stored as such, in a communication lexicon, together with their families of related senses, which originated as cases of pragmatically derived (*ad hoc*) senses but have become established, due to their communicative efficacy and frequency of use. Third, in an attempt to find empirical evidence for the proposed linguistic form-meaning divide, two very different cases of atypical linguistic and communicative development are considered: autistic children and deaf children who develop Homesign. The morpho-syntax (the formal side of language) appears to unfold in much the same way in both cases and is often not much different from that of typically developing children, but they diverge markedly from each other in their communication/pragmatics and their development of a system (a lexicon) of meaningful words/signs.

## 1. Introduction

Relevance theory (RT) provides a richly interdisciplinary framework for the investigation of communication and language, and it has been fruitfully employed by psychologists, philosophers, translation theorists and literary specialists, among others. It has, however, been criticized for its lack of interaction with core areas of linguistics, specifically work on linguistic structure (morphology and syntax) (Smith, 2019), a somewhat ironical situation, given that the theory typically finds its academic home in departments of linguistics. This paper, which focuses centrally on words and their meanings, continues the interaction of relevance-theoretic pragmatics with both philosophy of language and empirical psychology, while also suggesting how one aspect of its interface with the computational core of language might work.

In Section 2, I first look at the phenomenon of ‘*ad hoc* concepts/senses’ within relevance-based lexical pragmatics and a recent application of this notion in the philosophy of language; then, I move to a related but importantly different notion of ‘*ad hoc* words’ and the role of metonymy in their creation, ending with thoughts about the fundamental nature of the kinds of associative connections typical of metonymy, which appear to be basic and ubiquitous in our language use and in communication more generally. In Section 3, a distinction between language (construed in narrow linguistic/computational terms) and its communicative use is adopted, with words straddling the divide (having both morpho-syntactic structure and pragmatically-originated meanings). The main import of this section is to show how a syntactic treatment of words as phrasal structures, on the one hand, and the lexical pragmatic account of new word meanings, on the other, come together in explaining non-compositional word meanings (and indeed the very notion of a ‘word’). The section ends with thoughts about the kind of lexicon that sits best with this pragmatically-based view of polysemy. Section 4 is devoted to presenting evidence from two profiles of atypical linguistic and/or communicative development, which, with some provisos, points to the distinct and dissociable trajectories of the formal (morpho-syntactic) and the conceptual-semantic, thus further supporting the position that this constitutes a natural divide in human cognitive architecture. I end with a short discussion of the language ‘code’ (syntax and lexicon), which provides rich evidential input about the speaker’s meaning for the relevance-based pragmatics to work with.

## 2. Words: linguistic decoding and pragmatic inference

### 2.1. Lexical meaning adjustment and *ad hoc* concepts

From their earliest work on relevance theory, Sperber and Wilson (1986/1995) have drawn a fundamental distinction between a code model of communication and an inferential model, emphasizing that what a speaker means, what she intends her audience to grasp, when she produces a linguistic utterance is seldom, probably never, fully encoded in the linguistic meaning of the expression(s) employed. The stable established meaning provided by the linguistic components of the utterance typically (sometimes radically) underdetermines the meaning communicated. The insight comes from Grice (1967) and Donnellan (1966) in the first instance, but Grice seems to have confined pragmatic inference (‘conversational logic’, in his terms) to the recovery of a speaker’s implicit meaning (implicatures) while viewing the explicitly communicated meaning (‘what is said’) as essentially encoded.^1^ Since then, there has been much work in the RT framework that has demonstrated the role of pragmatic inference in contributing to the proposition explicitly communicated (termed ‘explicature’ in RT). This includes processes of disambiguation, saturation (e.g., assigning referents to pronouns and other indexical elements) and free enrichment (i.e., recovering components of meaning in the absence of any linguistic mandate to do so). The latter includes cases of ‘unarticulated constituents’ of propositional content (Carston, 2002; Recanati, 2002; Carston and Hall, 2017), but also cases where a linguistically provided meaning is pragmatically modulated so as to deliver a contextually relevant ‘*ad hoc*’ concept/sense for the word or phrase. I focus on the latter here; that is, cases where an established sense of a word or phrase is retrieved from the memorized store (the lexicon), as part of the linguistic decoding process, but is adjusted by relevance-based pragmatic inference.^2^

According to the RT lexical pragmatics account, the forming of an *ad hoc* occasion-specific meaning or sense for a word is a consequence of standard pragmatic processes of selecting contextual assumptions, drawing cognitive implications from the utterance in this context and making appropriate adjustments to the explicature. The ultimate result, an interpretation of the utterance, is a set of assumptions (taken to comprise the speaker’s intended meaning) which meet the criterion of optimal relevance and are in an inferentially sound relationship with one another. The pragmatic process of *ad hoc* concept formation may result in a narrowing of denotation, e.g., the use of ‘drink’ to mean alcoholic drink, or a broadening, e.g., the use of ‘flat’ to describe a surface that is relatively free of bumps, or various combinations of narrowing and broadening, e.g., ‘princess’ (its encoded meaning entailing royal parentage) used to denote a haughty, pampered, demanding young woman, and so including some non-royal women and excluding some actual princesses (the well-behaved ones). Some of these new senses for a word become sufficiently frequently used and widespread as to become established senses of the word; they are stored in the lexicon with the word’s other established senses and retrieved together with them when the word is accessed; in such instances, we have typical cases of ‘semantic polysemy’. However, many such *ad hoc* concepts/senses are merely occasion-specific and transient.

As an example, consider the word ‘mother’, which can be used in the following three ways (among others), to refer to (a) X’s biological mother, (b) X’s adoptive mother (legal but not biological mother), and (c) the person with whom X feels a special bond of reciprocal affection (who may not be X’s biological or adoptive mother, but someone who gave her the kind of nurturing that is normatively associated with a mother). Let us consider an RT account of how this third concept expressed by ‘mother’ might be recovered in comprehending the following utterance:

1. I owe so much to my aunty Jane – she was my real mother

Assume the word ‘mother’ encodes (i.e., has as a conventionalized sense) the atomic concept mother which provides a direct link to an ‘encyclopedic entry’ of assumptions/beliefs about mothers, including the following (and much more):

- A mother is a female parent [with further information about biological mothers, adoptive mothers, step-mothers, surrogate mothers, etc.].
- A mother is expected to provide the love and nurturing that ensures the child thrives physically and psychologically.
- A mother may be controlling and manipulative in ways that are not beneficial to a child.

Some elements of encyclopedic information are more accessible (more highly activated) than others, depending on the content of the rest of the utterance and the specifics of the occasion of use. For the current example, the most highly activated items of information are likely to be those in (b), which are then used as contextual assumptions/premises in deriving *cognitive implications* (e.g., Jane gave the speaker the love and nurturing expected of a good mother; this was highly beneficial to her physical and emotional development, etc.), which, in turn, via a mechanism of ‘mutual parallel adjustment’ of explicit content, contextual assumptions and cognitive implications, modulates the concept expressed/communicated by the word, yielding an *ad hoc* concept mother*, a concept whose denotation is both broader than the encoded concept mother, as it includes people who are not the female parent of a child but have given the child a kind of motherly nurturing, and also narrower in that it excludes negligent mothers (who are female parents). This inferential process stops when context-specific expectations of relevance (formed on the basis of the presumption of ‘optimal relevance’ conveyed by all utterances) are satisfied. The *ad hoc* concept/sense that is inferred is a constituent of the explicature of the utterance, taking the place of the decoded concept mother.^3^

The utility of this account in explaining cases of word meaning variation in other fields has been demonstrated recently by several applications in the philosophy of language. I focus here on one of these, as developed by Baumgartner (2022, 2023), who discusses so-called ‘dual character concepts’ (DCCs), that is, concepts that have both a descriptive dimension and a normative dimension. Standard cases discussed in the philosophical literature are ‘poet’, ‘artist’, ‘philosopher’, ‘scientist’, ‘friend’, ‘soldier’, ‘woman’, ‘man’. An attested case of the last of these is the statement ‘Hillary Clinton is the only man in the Obama administration’, where ‘man’ is clearly not being used descriptively (to mean ‘male, human, adult’) but normatively, that is, to pick out properties which, according to a (now largely discredited) social stereotype, are expected of a ‘real man’: psychological strength and courage, forcefulness, steadiness in the face of adversity, etc. (see Leslie, 2015 for extensive discussion of this example). The descriptive concept man and the normative concept man* expressed here are what the philosophers term ‘fully dissociative’ in that they set up two distinct categories: someone may be a man descriptively but not normatively (i.e., an adult male who lacks the normative properties of mental strength, courage, etc.), and someone else may be a man only normatively (as Hillary Clinton is claimed to be in the utterance above). An RT account of the latter concept man* would be essentially the same as that given above for mother*, using the social stereotype of a ‘real man’ to derive cognitive implications about the person so described, from which, in turn, the *ad hoc* concept is derived, a concept whose denotation is both narrower in some respects and broader in others than the descriptive concept (hence the noted dissociation between the categories they denote).

Most of those who have analyzed the DCC phenomenon take a semantic view, maintaining that those words which have this dual character (e.g., ‘philosopher’, ‘artist’, ‘scientist’, ‘mother’, ‘man’, ‘woman’) are cases of lexical polysemy, both senses being established across a population of users and stored in their mental lexicon (e.g., Leslie, 2015, p. 120). However, as Baumgartner notes, the virtue of the pragmatic account as given above is that it can explain a much wider range of cases than the lexical semantic view, which is restricted to those that have become conventionalized. It is certainly possible that some are now cases of semantic polysemy, e.g., ‘man’ and ‘mother’ in the normative senses discussed above, perhaps also ‘philosopher’ in the sense of a person who typically seeks answers to difficult questions about meaning or ethical issues via rational thought/argumentation, whether or not that person is a professional philosopher (Baumgartner, 2022). However, the pragmatic inferential account gives us both an explanation of how these established normative senses arose in the first place, and an account of cases that are not lexicalized and/or trade on normative values that are not public or established, but are themselves *ad hoc* and contextual. For a possible case of the latter, imagine the following: there is a family, the Hansens, the mother of whom emphasizes to her children that they should stay positive, calm and cheerful, even when difficult or upsetting things happen to them; while most of the family manage to comply with this ‘norm’ most of the time, the youngest child, Billy, tends to be moody and morose; next door lives his best friend, Joey Wilson, who is a happy-go-lucky boy. One day, Mrs. Hansen admonishes Billy, saying: ‘Joey is more of a Hansen than you are’, meaning, of course, that Joey, who is not a Hansen but a Wilson, has the (normative) characteristics of a Hansen family member: he is a Hansen*. As Baumgartner says, the lexical pragmatic approach (in terms of *ad hoc* concepts) can account for the full range of cases (whether *ad hoc* and transient, established and lexicalized, or somewhere in between), while the lexical polysemy account applies only to the lexicalized (conventionalized) cases.

My aims in this section have been: (a) to briefly describe the relevance-based account of *ad hoc* word meaning creation as a pragmatic contribution to utterance comprehension and a significant source of polysemy, and (b) to show, via exposition of Baumgartner’s work on dual character concepts, the potential utility of this account in helping to explain certain cases of multiple word meaning which are central to debates in the philosophy of language.^4^ In the next subsection, I move to a different kind of lexical pragmatic creativity, that is, the coining of new (*ad hoc*) words in the process of online communication/interpretation.

### 2.2. Lexical innovation: *ad hoc* words and the role of metonymic associations

New words coined on the fly in communication (as opposed to via offline stipulation) may take various forms, including (a) cases of standard word formation involving regular processes of affixation as in ‘detector-ist’, ‘expir-ation’, ‘burglar-ize’, ‘worst-est’; (b) blends, which take parts of two distinct words and form a composite, e.g., ‘brunch’, ‘motel’, ‘franglais’, ‘blizzaster’; (c) conversions, e.g., the verbs ‘to favorite’, ‘to laser’, ‘to lawn’, ‘to prodigy’ based on pre-existing phonologically identical nouns. There is a syntactic-semantic story to be told about how these new coinages acquire their compositional meanings and a relevance-theoretic pragmatic story to be told about how, for those that have them, they acquire non-compositional meanings. The syntax and pragmatics of the affixation cases are addressed in Section 3, but here I focus on ‘conversions’ (and specifically denominal verbs), which are distinctive in that there is no phonological difference between them and the nouns on which they are based.

Here is a sample of the phenomenon at issue: (2a)-(2b) are attested new(ish) *ad hoc* cases, (2c)-(2e) are more familiar, with two clearly distinct uses of the verbs in the (d/d’) and (e/e’) cases, and those in (2f) are fully established/conventionalized:

2. a. ‘I’m trying to *room* all the talks in the same building’ (conference organizer).
b. ‘Vasko Vassilev *prodigied* his way through the *Carmen Fantasia*’ (Alan Rickman).
c. ‘The prisoner *houdinied* out of the top security jail’.
d. ‘The factory *sirened* midday’.
d’. ‘The police *sirened* the Porsche to a stop’.
e. ‘The boy *porched* the newspaper’ [threw X onto a porch].
e’. ‘The developer *porched* all the bungalows’ [added a porch onto X].
f. to hammer (a nail, a box flat), to shell (walnuts), to starch (shirts), to dust (the corners of the room; the cake with cinnamon); to treasure (our time together); to bike, to bus, to jet; …

On a traditional linguistic view, conversions are cases of derivational morphology, essentially the same as the move from the noun ‘standard’ to the verb ‘standard-ize’, or from ‘code’ to ‘cod-ify’, but with a zero (phonologically empty) affix. However, advocates of this view have often noted, with some unease, the extensive range of meanings that conversion verbs can have, meanings that are unsystematic and unpredictable, unlike that of typical cases of affixation. Consider, for instance, the very different kinds of interpretation (and relation between verb and parent noun) of ‘to room’, ‘to prodigy’, ‘to porch’, ‘to siren’, and ‘to dust’. In their ground-breaking study of nouns ‘surfacing as verbs’, Clark and Clark (1979) treated them as cases of *lexical innovation*, new words coined on the fly in communication, whose meanings are highly context-sensitive, with only the very general linguistic constraint that they are verbs.

So these spontaneously coined denominal verbs require a pragmatic explanation, in which the encyclopedic information which comes with the parent noun, e.g., about porches in the cases of (e) and (e’), plays a key role, along with readily accessible contextual information, e.g., about boys delivering newspapers or developers building houses for (e) and (e’). In his study of conversions (both noun to verb and verb to noun), Bauer (2018) takes this pragmatic account one step further, maintaining that they are *metonymic shifts* made by speakers in communication. As he puts it, they are typical of figurative interpretations in being ‘unpredictable and unrestricted’ (Bauer, 2018, p. 180) and the relations between the meanings of the parent noun/verb and the derived verb/noun are typical of metonymic associations, e.g., *location for action/event* as in ‘*porch* the newspapers’, *attribute for behavior* as in ‘*prodigy* the *Carmen Fantasia*’, *person for behavior* as in ‘*houdini* out of the cell’, *instrument for action* as in ‘*siren* the car to a stop’.

However, there is a notable departure here from standard cases of metonymy, as reflected in the following definition of metonymy: ‘a figure of speech involving substitution of the name of an associated attribute or adjunct for that of the thing meant’ (OED). That is, metonyms typically involve the use of a noun to refer to an associated entity, person or thing rather than to an action or process, and so *do not involve a change of syntactic category*. For instance, ‘a farm hand’, ‘the city suits’, ‘the crown’, ‘Downing Street’, ‘the ham sandwich’, and a wide range of semi-regular cases: e.g. *container for contents* (e.g., ‘He drank the whole bottle’); *creator for work* (e.g., ‘I’ve read Dickens’); *place for event* (e.g., ‘Waterloo’, ‘Vietnam’, ‘Woodstock’); *animal for meat*, etc. The relation is often described as one of ‘contiguity’ (spatial, temporal or casual/resultative) between *things* in the world (distinguishing it from other cases of non-literal use: resemblance for metaphor and antonymy for irony).

Conversions do not seem to fit this standard definition of metonymy, and more generally, figures of speech (e.g., hyperbole, metaphor, irony) do not usually involve a change of word category (creating a verb from a noun, or vice versa). It might seem then that in these standard cases of nominal metonymy, e.g., ‘hand’, ‘suit’, Downing Street’, ‘ham sandwich’, what we have is just another instance of *ad hoc* concept construction, as discussed in the previous section, where the word or phrase is given a new meaning (which may become established over time giving rise to semantic polysemy). However, in recent work within relevance theory on these standard cases of nominal metonymy, Wilson and Falkum (2015, 2020) have argued that, in fact, ‘metonyms arise as motivated neologisms’ i.e. metonymic uses are spontaneous pragmatic processes of *new word coinage.* On their pragmatic account, the new word (specifically its meaning, as its phonology is a given) is inferred from accessible information in the encyclopedic entry of the input noun (e.g., ‘suit’, ‘hand’, ‘ham sandwich’) and information in the wider discourse context, guided by the prevailing relevance-theoretic comprehension procedure. Importantly, they maintain that this is different from meaning modulation (narrowing/broadening/metaphor) because the encoded concept/sense (e.g., hand, suit, ham sandwich) and the new *ad hoc* (metonymic) concept/sense (e.g., hand*, suit*, ham sandwich*) do not share cognitive implications and their denotations are disjoint; the output is thus a different (*ad hoc*) word from the input word.^5^

What we see here is a nice convergence of independent work: Bauer’s (2018) claim that conversions (new words made from existing words, e.g., denominal verbs) are instances of metonymy and Wilson and Falkum’s (2015, 2020) position that standard nominal metonymies are motivated word coinages (denominal nouns). Putting these together, what we get is the view that when an existing word is used (without affixation or any other phonological change) to convey a metonymically associated meaning (or to refer to an associated entity/action/process in the world) a new word is thereby created, which may or may not involve a syntactic category change.^6^^,^^7^ This applies equally to words that are more transparently complex because of their affixations, so, e.g., ‘transmission’ with its meaning of car’s gearbox looks like a case of *process for instrument* metonymy and ‘reading’ with its meaning of an interpretation (as in ‘His reading of the text was more allegorical than mine’) is a *process for result* metonymy. If Wilson and Falkum are right, these are new words, distinct from the words ‘transmission’ meaning the process of transmitting and ‘reading’ meaning the process of reading, so they are further instances of denominal nouns. These morphologically complex cases are discussed in more detail in Section 3.1.

A worry about metonymy as a means of using existing words to generate new words/senses is that it seems to be very general and unconstrained, allowing users to take a word and form a new (phonologically identical) one whose meaning/content is in some sort of salient associative relation with the meaning/content of the existing one; as long as a speaker can be more or less sure that the association (spatial, temporal, resultative) is apparent to her audience, she is free to create the new word. However, metonymy just does seem to be an easy basic conceptual/pragmatic process. It arises spontaneously and cross-culturally very early on in children’s communicative use (even pre-linguistically) and in their comprehension (well before they can comprehend metaphor) (Falkum, 2019; Köder and Falkum, 2020; Wilson and Falkum, 2020). Experiments testing people’s appreciation of well-established polysemies find that they consider metonymically-related senses to be more closely related than cases involving ‘resemblance’ (narrowing, broadening, metaphor) (Klepousniotou and Baum, 2007). In fact, there is less ‘semantic overlap’ (i.e., sharing of features/properties) in metonymy than in the resemblance cases, related by pragmatic modulation, so the apprehended ‘relatedness’ must simply reflect the strength of the associative connection in people’s minds. Klepousniotou et al. (2008) assume there must be some sort of ‘core meaning’ shared by established metonymies like the animal-meat (e.g., ‘lamb’) and institution-building (e.g., ‘school’, ‘church’) cases and that these are all ‘literal’ (rather than figurative) uses of the words involved (for discussion, see Carston, 2021). Whatever one may think of these assumptions, they indicate that metonymic associations (spatial/temporal/resultative contiguities in the world as apprehended by us) are quite psychologically basic. Although metonymy is usually seen as less interesting (and certainly less beautiful) than metaphor, it may be that, in certain ways, it is more fundamental to our cognitive and communicative lives.^8^

Note that a significant consequence of the view that metonymic conversion is a means of creating new words is that polysemy must cross syntactic categories, that is, a family of closely related senses may be spread across nouns and verbs, e.g., ‘porch’, ‘starch’, ‘houdini’, ‘jet’, ‘prodigy’, and even across nouns/verbs/adjectives, e.g., ‘stone’, ‘back’ (Carston, 2019). What these words share is their phonology and, more crucially (since homonyms also share phonology), a *root*, which can be notated as follows: √stone, √porch, √houdini, √prodigy, etc. So, it is really roots rather than words that track polysemy (i.e., families of interrelated senses). Yet words seem to be highly salient to ordinary language users (Julien, 2007), and it is words, rather than roots, that are employed as our minimal communication units (one-word utterances) and are logged in our pragmatic lexicons (see Section 3.3). Some current syntacticians maintain that *words have no status in the grammar*, so, e.g., ‘nationalize’, ‘solidarity’ are phrasal structures, as are ‘siren’, ‘porch’, and even apparently simple words like ‘cat’ and ‘run’ (according to Marantz (1997), Borer (2015), Harley (2014), and many others), for which the basic elements are roots (and functors, including categorizers). As Acquaviva (2022, p. 283) says: root-based syntax ‘may model the distinction between polysemy and homonymy in formal terms, so only polysemous words share the very same syntactic root’, so, e.g., there is a single root for the noun/verb/adjective ‘stone’, with their pragmatically interrelated meanings, but two roots for homonyms like ‘bank’ and ‘bug’, which have two unrelated families of meanings. The syntactic side of conversions and of other new words, and the importance of roots, are discussed further in the next section.

## 3. The language faculty and ostensive communication

### 3.1. ‘Words’: syntax and pragmatics

According to the root-based approach to syntax, touched on above in the discussion of conversions and cross-categorial polysemy, words have no formal or theoretical status in the grammar; they are phrasal structures and so, like all phrasal structures, they have a compositional semantics, which is a function of the meanings of the basic parts and their structural combination. On some views (e.g., Panagiotidis, 2014; Borer, 2017), a root is nothing more than an index or address tracking its occurrence across categories, so it is meaningless as well as categoryless; only once it has been conflated with a syntactic categorizer is it assigned a meaning, e.g., [_N_ √form], [_V_ √houdini], [_Adj_ √stone]; this is the first level of content, on the basis of which the compositional meaning of a more complex word (e.g., ‘formation’, ‘stonily’, ‘adorable’, ‘houdinify’) is generated.

However, as widely noted, there is a glaring issue for this single syntactic engine approach to word structure: many of those phrasal entities that we think of as words have a *non-compositional (idiosyncratic, unpredictable) meaning*. Examples abound: ‘reactionary’ meaning backward-looking, ‘transmission’ meaning gearbox of a car, ‘flakey’ meaning unreliable, ‘execution’ meaning state-sanctioned killing, ‘demonstration’ meaning organized mass protest, ‘naturalize’ meaning make someone a citizen of a country, ‘liquidate’ meaning kill someone (violently), ‘recital’ meaning solo concert … There are two points to note here: (a) Each of these words also (inevitably) has a compositional meaning, although there is considerable variation in the current usage of these meanings (e.g., while the compositional meaning ‘transmit + tion’ is widely used, the compositional meanings ‘recite + al’ and ‘reaction + ary’ are much less so); (b) The non-compositional (idiosyncratic) meanings are not completely unrelated to the corresponding compositional meanings (or to the meanings of other words with same root). What lies behind this relatedness is, as already noted in Section 2, the fact that the very same pragmatic processes of meaning/sense modulation and metonymic word coinage, typically discussed within relevance theory only with regard to monomorphemic words, apply equally to these more structurally complex cases, a point exemplified further below.

The key issue here for the syntactic treatment of word structure is that it does not account for the non-compositional meanings that complex words can have. Of course, the advocates of this approach to word structure are well aware of the issue and some have developed explanations for *why* and *when* non-compositional meaning is possible, although not for the particular meanings that arise (which, I maintain, is the job of pragmatics). Their general idea is that there are specific ‘syntactic domains’ within which non-compositional (atomic, idiosyncratic, special, unpredictable) meaning/content can emerge. So ‘recital’, ‘naturalize’, ‘reactionary’ have a specific *kind* of syntactic structure which, although it has a compositional meaning (like all syntactic structures), allows for (but does not require) assignment of a special (non-compositional) meaning. I cannot begin to assess here the relative strengths and weaknesses of the various proposals, which are often highly technical and developed within different syntactic frameworks. What follows is a very simplified indication of one of the best-developed accounts, that of Borer (2013a,b, 2014).

Borer’s syntax is a ‘constructionist’ theory: the grammar generates syntactic templates (event structures) into which roots are inserted, e.g., √dog or √stone is inserted into a structure within which it becomes noun-equivalent, verb-equivalent, or adjective-equivalent, depending on its position within the structure. As well as roots, there is another kind of basic element in the system, namely ‘functors’ (which include tense, aspect and number indicators, determiners, and categorizing affixes such as ‘-ize’, ‘-ary’, ‘-tion’, etc). Borer distinguishes two different kinds of functors and these play crucially different roles in her account of syntactic domains of non-compositional meaning (or Content, as she calls it). These are (1) *C-functors*, i.e., categorizers, e.g., *n*ominal, *v*erbal, *a*djectival, which may (but need not) be realized phonologically by various affixes, and (2) *S-functors*, which project further levels of structure; these include the determiners (e.g., ‘the’, ‘those’), count/mass and number (singular/plural) indicators for nominal structures, and tense and aspect for verbal structures.

Categorizers (C-functors) allow non-compositional meaning assignment at multiple levels, so, for instance, in the structure ‘the [{([√nature _N_] al _A_) ize _V_} ation _N_]’, Content can be assigned at each of the structural domains headed by N, V, or A, and, as noted above, the domain delimited by V here has, in fact, received a non-compositional (idiosyncratic) meaning/content: make someone a citizen of a country. Structures headed by S-functors do not allow this:

3. a. Tense phrases: ‘jump-ed’ – meaning must be compositional.
b. Number phrases: ‘book-s’ – meaning must be compositional.
c. Determiner phrases: ‘the/that/my book’ – meaning must be compositional.
d. AS-nominals (which inherit the *A*rgument *S*tructure of the verb from which they are derived): e.g. ‘destruction’ (of the city by the barbarians in a single day), ‘teaching’ (of the physics class by Mary).

C-functors indicate structure points at which Content (that is, non-compositional meaning) can be assigned. On Borer’s (2013a, 2014) account, these are points at which there is a search of what she calls the ‘Encyclopedia’ for a matching content. The ‘Encyclopedia’ is not a component of the grammar, but rather lies within the Chomskyan conceptual-intentional (semantic-pragmatic) systems with which the syntactic engine interfaces, and it is the locus of stored non-compositional meanings. It is akin to (though by no means identical to) my conception of the communicative lexicon, discussed below in Section 3.3.

A striking piece of evidence in support of Borer’s account comes from the behavior of two different kinds of verb-derived nominals, examples of which have already been briefly mentioned: those that can take a non-compositional meaning, e.g., ‘transmission’, ‘recital’, ‘referral’, ‘revolution’, ‘solicitor’ (known as R-nominals), and those that cannot, as in 3(d), (known as A-S nominals). To make this clearer, here are instances of the two kinds of case, where each member of the pair, (a) and (b/c), has been derived from the same verb and is phonologically identical:

4. a. The transmission of news from Ukraine by the BBC (for several hours…).
[*cf.* The BBC transmitted news from Ukraine for several hours.]
b. The car’s transmission [= gearbox] is in good condition.
c. * The car’s transmission by Nissan for several years …
[cannot mean: *the transmission (= gearbox) as made* by Nissan for several years].

5. a. The referral of Mary by her doctor to a rheumatologist.
[*cf.* The doctor referred Mary to a rheumatologist].
b. The referral [= person referred] left the consultant’s room feeling reassured.
c. * The referral by her doctor to a rheumatologist [has arrived for her appointment].

In each of these cases, the (a) version has inherited the argument structure of the verb from which it is derived and its meaning is compositional, while the (b) version has a non-compositional meaning and it does not allow the verbal arguments, as shown in (c), in each case. That is, we find a correlation of properties here: *R-nominals* can have special (non-compositional meaning) and do not take argument structure; *AS-nominals* have argument structure (whether explicit or left implicit) and cannot have special (non-compositional meaning). Members of each pair are derived from the same verb, [transmit], [refer], and are phonologically identical, so are distinguished by syntax alone. Here are their syntactic structures (simplified):

6. R-nominals (can have non-comp meaning): (_N_ -tion [_V_ transmit])
AS-nominals (cannot have non-comp meaning):
[_N_ -tion ([_F2_ subj [_F1_ obj [_V_ transmit]]])].

The R-nominal is headed by a categorizer with no intervening S-functors, so marks out a structural domain which allows assignment of Content. The AS-nominal, on the other hand, contains what is abbreviated here as functional structures F1 and F2 (the subject/object arguments, e.g., ‘the BBC/the news’, ‘the doctor/to a rheumatologist’), so is replete with S-functors, which block assignment of Content (non-compositional meaning).

Assuming, then, that Borer’s account is well-grounded, a hypothesis, whose confirmation would be very pleasing for the picture I am drawing here, is that the syntactic structures defined by these domains are typically what the language user perceives as ‘words’ and which can, therefore, be the basis of the kind of pragmatic lexical modulation processes that were discussed in Section 2. This seems plausible but needs empirical support. If it proves to be right, these domains provide the necessary link between the formal computational system and what I call the pragmatic or communicational (user-based) lexicon.

To end this section, let me indicate, with some more examples, the ways in which the non-compositional meanings of some of the morphologically complex words discussed above mesh with the pragmatic account of meaning modulation (narrowing/broadening) and new word coinage. I leave the specifics of plausible contexts for these meaning creations to the imagination of the reader. The verb ‘naturalize’ with the non-compositional meaning naturalize (= make a foreigner into citizen of a country) is a pragmatic narrowing of the more general compositional meaning [natural + ize] roughly paraphrasable as ‘to make natural’. Such narrowings are common in specific contexts in which jargon terms arise: e.g. ‘transformation’, used for a kind of grammatical operation in linguistics, and ‘transference’ used in psychoanalysis for a particular psychological process, both narrowings of the general compositional meaning of the structures involved. These are both R-nominals (and have AS-nominal counterparts). Something a bit different is going on with the following cases suffixed by ‘-ing’: ‘reading’ with the non-compositional meaning reading (= an interpretation), as in ‘His reading of the novel was highly allegorical’, and ‘teaching’ with the non-compositional meaning teaching (= a set of ideas/a lesson), as in ‘She was profoundly influenced by Buddhist teachings’. These seem best analyzed as involving a *metonymic shift*, given that arriving at an interpretation of a text is typically a *result* of a process of reading (= read + ing) that text and a set of ideas is typically a *result* of someone’s teaching (= teach + ing) them, a standard metonymic relation, according to Bauer (2018), and therefore, if the account of metonymy given in Section 2 (Wilson and Falkum, 2015, 2020) is right, these are new words, new communication units for users. In the case of ‘reading’, there appears to have been also a broadening of meaning in that one can have a reading not only of texts but also of situations and people: e.g. ‘On my reading of the situation, we are doomed.’ Furthermore, the verb ‘read’ itself seems to have acquired this meaning of ‘interpret’: ‘As I read the situation, we are doomed’, perhaps by some sort of back-formation process, and thus a compositional meaning of ‘reading’ (= interpretation) is reinstated. Finally, a similar sort of analysis of the non-compositional meaning of ‘transmission’ (= car’s gearbox) can be given: a *narrowing* of denotation (to the specific kind of transmission that takes place in the engine of a car) and a metonymical transfer to the object responsible for this specific kind of transmission (the gearbox), creating a new word or communication unit for language users. For more detailed discussion, see Carston (2022).

Summing up: what I hope to have shown here is that a root-based syntactic account of word structure with C-functor defined domains of Content can be integrated with the relevance-based pragmatic account of how specific non-compositional meanings (atomic Contents) of words arise (by meaning modulation and metonymic transfer) to give a full and unified account of word meanings. The account applies equally to the overtly affixed cases discussed in this section, to so-called ‘conversions’^9^ such as the verbs ‘porch’, ‘houdini’, ‘prodigy’, and to seemingly simple words such as the noun ‘cat’, the verb ‘put’ and the adjective ‘red’.^10^

### 3.2. Language faculty: narrow/broad; generative/stored; individual/social

According to Hauser et al. (2002), the broad folk notion of language is a mosaic of components, and fruitful investigation requires carving up this broad conception into tractable domains of study, separating out “questions concerning language as a communicative system and questions concerning the computations underlying this system, such as those underlying recursion.” (Hauser et al., 2002, p. 1567). Generative linguists in the Chomskyan tradition focus on the latter, the narrow internal linguistic system (I-language), that is, syntax and its interfaces with conceptual-pragmatic capacities, on the one hand, and perceptual/articulatory systems, on the other. This computational system is not essentially an instrument of communication, although, as a matter of fact, it is widely and productively employed in communication, as enabled by its interfaces. Most psychologists, on the other hand, focus on language as a communication system, investigating the perceptual and cognitive processes that take place when we produce and comprehend linguistic utterances.

Words have a sort of double status, as they straddle the linguistic/communicative divide: they are phrasal entities generated by the syntax (language narrowly construed) – there are no words without syntax – but they are also salient as basic communicative units with shared meanings, some of which become conventionalized and so enter into the overlapping lexicons of particular groups of communicators, making them a socio-cultural component of language broadly construed. While formal linguists are interested in words as syntactic entities (with phonological and semantic properties), psychologists tend to focus on them as communicative units, investigating their activation, their retrieval/recognition, and their integration with other words in the course of utterance processing, often measuring the time course of their online production and comprehension.

These very different stances are more a matter of preferred focus than of incompatibility or rival positions – of course, we want an account of language as a faculty of the mind *and* an account of how it works when used in communication. However, the two different orientations can become incompatible (even antagonistic) when the discussion moves to the fundamental nature of language, what it is for, and its evolutionary origins. Some psychologists maintain that language just *is* a communication system and that it should be investigated as such, and be taken to have evolved (been selected) for that purpose. For instance, Vigliocco et al. (2014) decry: ‘the (explicit or implicit) assumption that the object of investigation – language – can be properly and sufficiently addressed by ignoring other characteristics of face-to-face interactions: the communicative context in which language has evolved, in which it is learnt by children, and in which it is most often used’, maintaining that language must be studied as a multimodal mix of speech, prosody, gesture and facial expression. This is, of course, directly at odds with the generative linguistic stance according to which the core property of human language is its recursive syntax, which is taken to be an entirely proper study in itself (Hauser et al., 2002; Berwick et al., 2013).

The ‘purpose’ of language and its evolutionary origins similarly divides these groups. Those with a communication-orientation maintain, albeit with important differences of detail among them, that it emerged in order to fulfil social-cooperative-communicative needs specific to humans (Tomasello, 2008; Vigliocco et al., 2014; Scott-Phillips, 2015). The syntax-oriented theorists point out that linguistic structures, with their gaps and long-distance dependencies, are not optimized for communication but rather for computational ease in thought (Chomsky, 2010), and that some easily interpretable structures are glaringly ungrammatical (e.g., ‘Who did John call Mary and --’). On this ‘biolinguistic’ view, language (i.e., the capacity to recursively combine concepts) arose quite suddenly in the species via a rewiring of the brain, which conferred considerable fitness-enhancing advantage on the individual so endowed, enabling complex thought, understanding, and planning, a capacity then transmitted to offspring, and so coming to predominate. On this view, the use of language for communication is a subsequent and secondary development, a matter of linking the core linguistic capacity to sensorimotor systems required for its externalization (including the property of linearization, arguably not necessary for thought) for verbal utterance production and perception/comprehension.^11^

Where are relevance theorists situated in this debate? As pragmaticists, their focus is, of course, on communication, and on linguistic meaning as providing evidence of what the speaker intends to communicate, rather than on detailed investigation of the formal properties of language. However, there appears to be something of a divide between those relevance theorists who see language as a system whose raison d’être is communication, having evolved as an instrument of a pre-existing ostensive communicative capacity, which created a particularly favorable environment for the emergence of language (Sperber, 2000; Sperber and Origgi, 2010; Scott-Phillips, 2015), and those who find the Chomskyan story more promising, that is, that language (understood as the core recursive computational system) first effected a transformative change in our powers of thought, only secondarily being externalized and used in communication (Carston, 2015, 2023; Reboul, 2017). This a fascinating and complex area, which I cannot pursue further here, but in this paper I am assuming the latter position. The next section addresses words as conventionalized communicative units, that is, as components of language broadly construed.

### 3.3. The communicational lexicon and polysemy

The way words work in our cognitive and communicative lives is very different from the way syntax works. First, as discussed in previous sections, word meanings are flexibly manipulated/adapted and new words are fashioned from existing words in order to express new concepts in ever-evolving contexts of communication. There is nothing comparable to this in the realm of syntax. Second, we continue to acquire new words throughout our lifetime, while our native language syntax is essentially in place and fixed by the age of five or six. Admittedly, young children learn words at a remarkably fast rate (several per day at the peak of acquisition), but they are also learning vast numbers of new facts at the same age. In line with this, the evidence indicates that this acquisition process is not achieved via a dedicated cognitive system as is the case for syntax, but rather via more general cognitive processes of learning and memory which are also employed in the acquisition of new facts.^12^ For instance, Markson and Bloom (1997) report studies in which children aged 3.7 and 4.3 were (like adults) as good at learning and remembering an arbitrary linguistically presented fact about a new unfamiliar object (e.g., ‘My uncle gave it to me’) as they were at remembering its name (e.g., a new word ‘koba’), and this was so even when the new arbitrary fact contained a novel word (e.g., ‘It came from a place called Koba’).^13^

As noted in the previous section, although words are syntactic entities, they have no privileged status in the narrow language faculty (syntax and its interfaces); they are simply one of the many phrasal structures generated by the syntax, whose basic units are roots and functors. For the ordinary language user, however, words are highly salient as basic units of communication^14^ and are stored in a lexicon, from which they are retrieved (with their families of related senses) in linguistic communication and comprehension, along with other linguistic phrases that have become well-established (conventionalized) and are accessed as a whole: e.g. idioms such as ‘spill the beans’, ‘trip the light fantastic’ and frozen forms such as ‘in cahoots with’, ‘kith and kin’. An individual’s communicational lexicon is a result of her communication history and consists of phonologically spelt-out forms and conceptual meanings, which can accrue many cultural/personal associations. This lexicon is a *performance system*, in Chomsky’s terms; it is a component of the language faculty only on a broad construal, lying outside the narrow linguistic ‘competence’ system and arising from socio-cultural processes of communication and conventionalization; it registers properties of words (and their senses) like frequency of use, which are irrelevant to the syntactic system, but are reflected in the processes of word recognition, retrieval, priming and comprehension measured in online psycholinguistic experiments.^15^

If the relevance-based pragmatic story told in the earlier sections is right, new *ad hoc* word senses/meanings are being fashioned in context all the time (via the concept modulation process that results in more specific and/or broader senses) and new *ad hoc* words are being coined (via metonymic associative processes), but only some relatively small subset of these becomes sufficiently well-established so as to enter a user’s mental lexicon. We need an account of the socio-cultural process(es) that result in the conventionalization of words and senses (setting aside those that come into being via authoritative stipulation); words as communication units (rather than syntactic entities) are, arguably, cultural phenomena and so are to be explained in similar terms as the evolution of other stable cultural items. The challenge here is to explain how the language use of individuals leads to group or population-wide communicative conventions. This is not an issue I can pursue here, except to mention briefly the interesting ‘epidemiological’ framework initiated by Dan Sperber. According to this approach, cultural phenomena, including words, are to be explained as the cumulative effect of multiple processes taking place within and between individual members of a population, that is, causal chains of mental and public representations, imperfectly copied from one token to the next but sufficiently similar to constitute a recognizable and stable type (Claidière et al., 2014).^16^

There are many questions about the nature of the entries for words in this communicational lexicon; focusing on the meaning side, I assume a word (that is, a phonological form classified as a noun, verb, adjective, etc. but with no more syntactic information than that) includes its family of related (established) senses. Most words are polysemous: consider, for instance, the verb ‘run’ in ‘run a mile’, ‘run a business’, ‘run a meeting’, ‘run for president’, the noun ‘line’ in ‘a line on a page’, ‘a line of a face’, ‘a line of washing’, ‘a line of work’ and the adjective ‘shallow’ in ‘shallow water’, ‘shallow valley’, ‘shallow bore’, ‘shallow thought’, etc. As noted by Wittgenstein long ago, in a discussion of the word ‘game’, there is no common definitional core (contrary to some current claims in the psychology literature) to these related senses; they are the result of chains of pragmatic inference, which take senses off in various directions dependent on the contexts in which they arise. It may be that the best way to think of how these sense families are represented is as a network of connected nodes, with proximate nodes representing closely related senses, and nodes separated by multiple nodes much more distantly related (Langacker, 1991; Recanati, 2017). Note that homonyms (e.g., ‘bank’, ‘bug’) will comprise two distinct entries in the lexicon each with its own network of related senses.^17^

The same account applies to noun-verb ‘conversions’, with their metonymically-derived non-compositional meanings, e.g., ‘dust’ (remove dust), ‘dust’ (sprinkle (e.g., sugar on a cake)), ‘porch’ (throw something on a porch), ‘porch’ (add a porch to a building), ‘houdini’ (make an incredible escape), and to other morphologically complex words, e.g., ‘reactionary’, ‘naturalize’, ‘recital’, ‘transmission’, ‘detectorist’, ‘flakey’. They appear in the communicational lexicon with their non-compositional (atomic, idiosyncratic) senses. As noted in Section 3.1, they each also have a compositional meaning, composed from the sense assigned to their smallest domain of content, e.g., [√nature + n], [√recite + v], [√detect + v], plus the further levels of categorization in their structure. This compositional meaning can be seen as a function of syntax and the relatively rigid semantics of the affixes (‘-ary’, ‘-ize’, ‘-ist’, etc.), so it is predictable, not idiosyncratic, and on those grounds need not be listed in the lexicon. However, if that meaning has become conventionalized (do compositional meanings ever become conventionalized?), it might be listed along with the non-compositional meanings of the word; this remains an open question, not to be decided simply on grounds of theoretical economy (Carston, 2021, 2022). Either way, we have here further cases of polysemy, whose source is a combination of syntax and pragmatics.

What then is the language code, what is it that is decoded (albeit by a process of ‘linguistic inference’, according to Fodor, 1983 and Sperber, 2018; see footnote 2)? Its outputs seem to be mental representations which are a product of both the narrow language faculty (syntax) and components of its interfaces, of which what I call the communicational lexicon is most relevant here. Creative use and pragmatics (= interpretive inference) are what we do with these decoded outputs. That is, from the point of view of utterance comprehension, the code consists of two parts: syntax (the narrow ‘linguistic’ faculty) and lexicon (a part of our conceptual-intentional or semantic/pragmatic systems).

As already noted, the communicational lexicon is a component of the language faculty only as broadly conceived; it lies outside the formal computational linguistic system and is a store of communication units with non-compositional meanings. The form-meaning (syntax-pragmatics) divide emphasized in the previous sections, is real and can lead to dissociations in children’s development, as shown by quite a wealth of empirical evidence, some of which is briefly surveyed in the next section.

## 4. The language-communication divide: empirical evidence

### 4.1. Autism and the form/meaning (syntax/pragmatics) divide

It is widely agreed and robustly attested that autistic children^18^ are impaired in their social interactions and in communication/pragmatics (see, e.g., Tager-Flusberg et al., 2005; Kissine, 2021), while the state of their specifically linguistic abilities is much less clear. Given the pragmatic account of (non-compositional) word meanings presented in this paper and in more detail in Carston (2022), and the more general thesis of a divide between the formal (morpho-syntactic) and the conceptual (word meaning) components of language, several predictions or hypotheses about autistic people’s abilities with words seem to arise: (a) inferring new, *ad hoc* (non-compositional) meanings in context is likely to be difficult, especially if the word already has an established meaning in the autistic person’s lexicon, and so (b) their lexicons will contain little polysemy (i.e., networks of established related senses), while (c) the formal syntactic aspects of words may pose little difficulty, that is, acquisition of complex structures like ‘formation’, ‘amplifier’, ‘demonstrate’, ‘transmission’.

Although it must be acknowledged that the evidence is patchy and any conclusions are tentative at best, I will survey some studies of autistic children that I believe point in the direction of a dissociation between their acquisition of formal aspects of language, on the one hand, and their grasp of conceptual word meanings and building of a communicative lexicon, on the other. As outlined in earlier sections, my general thesis is that the meaning components of our mental lexicons are fundamentally a product of communication, with many now established meanings of words having originated from processes of online pragmatic inference which take as input an existing word meaning and derive a new (*ad hoc*) contextually relevant meaning; this is a major source of the widespread polysemy of the communication units we deploy. If this is right, we might reasonably expect autistic children (and youth), who are known to have difficulty with flexible pragmatic word use, to exhibit concomitant difficulties in building a communicative lexicon, at least one that resembles that of typically developing (TD) children.

In a recent experimental study, Floyd et al. (2021) report that ‘children on the autism spectrum are challenged by complex word meanings’ (p. 2543); more specifically, they showed that autistic youngsters (aged 7–14) were shown not to have the facility with polysemy that TD children have. While the latter find it significantly easier to learn multiple related meanings of a word (polysemy) than to learn multiple unrelated meanings for a single word form (homonymy), the autistic group showed no difference between the two conditions. Floyd et al. conclude that polysemous words present a challenge to autistic children, and that they may benefit from interventions designed to help them ‘to recognize that a word witnessed in a particular context with a particular meaning can also be used in a different context with a related but distinct meaning.’ (Floyd et al., 2021, p. 2547).^19^ These results and the conclusions drawn from them mesh well with the account of the communicative lexicon that I have given, from which a prediction of difficulty with building polysemy into word meanings follows from a more general difficulty in allowing for the kind of flexibility of word meaning required to form contextually relevant on-the-fly *ad hoc* word meanings.^20^ However, there was, of course, no comparison being made here with these children’s formal linguistic abilities, so we need to look elsewhere to see if there is support for the position that the development of grammar may follow a different trajectory, coming from what is, in effect, a different source (the narrow computational linguistic capacity).

A comparison of this sort is what a longitudinal study by Naigles and Tek (2017) and Naigles (2022) set out to achieve. They studied early language development in autistic children (with an average starting age of 34 months) over a period of two years, examining their grasp of both formal/syntactic and conceptual/lexical aspects of language. Based on both a review of existing literature on these issues and their own findings, they propose that ‘the social difficulties of children with ASD lead the meaning-related components of their language to be relatively more impaired than the form-related components.’ (Naigles and Tek, 2017, p. 1), summing up their observations in the slogan ‘form is easy – meaning is hard’.

With regard to grasp of linguistic form (morpho-syntax), they report that the preschool-aged autistic children in their study were able to: (a) add appropriate plural markers to novel (nonsense) nouns, e.g., ‘wug’, and past tense markers to novel verbs; (b) map novel verbs in transitive frames onto causative rather than noncausative actions; (c) understand SVO word order (‘the girl tickled the boy’ vs. ‘the boy tickled the girl’); (d) understand wh-questions; (e) understand aspectual differences (e.g., ‘she’s picking the flowers’ vs. ‘she picked the flowers’). So, apart from some delay, these children manifested no significant difference in these areas from typically developing (TD) children, despite the fact that they engage in far less talking and other communicative interactions, so their spontaneous language production is much lower than that of TD children.^21^

Moving now to these children’s lexical semantic abilities (or, in my terms, their grasp of word meanings and the organization of their lexicons), this is where they seem to differ markedly from TD children. Some of the findings reported in Naigles and Tek (2017) and Naigles (2022) directly pertain to the children’s lexicons, while others seem to be more a matter of the kinds of concepts they form, which is, of course, likely to impact on the nature of their word meanings. Regarding the lexicon, they report that specific word classes, e.g., mental state verbs such as ‘think’, ‘know’ and ‘imagine’, and words referring to emotions are significantly less present in children with autism than in TD children. This is probably not too surprising, given the well-documented autistic difficulties with social cognition (or theory of mind). A second more telling difference is that when extending a novel label for an object (e.g., ‘dax’) to further objects, the autistic children appeared not to have the shape bias typical of TD children (i.e., shape of an entity is typically taken to be an indicator of its kind or class, rather than color or size or texture). Some of the autistic children generalized a word’s denotation on the basis of color, others required that entities have multiple properties in common. Thus, the denotations of the autistic children’s words for objects are likely to be idiosyncratic, often narrower than those of TD children.

Third, their categorical induction is impaired, as compared with TD children, that is, the ability to attribute a property (e.g., ‘eats grass’) which has been established for one instance of a kind, say, a rabbit, to other instances of the same kind (i.e., creatures falling under a word known to the children, here ‘rabbit’). This seems to be a matter of their understanding of natural kinds, which must affect the kind of encyclopedic information they incorporate in their concepts of these kinds and so may impact on their word meanings (assuming their words encode these concepts). Fourth, high-functioning autistic children were significantly less able to provide prototypical exemplars for a category word (e.g., ‘bird’, ‘flower’, ‘furniture’, ‘game’) than language-matched TD children. In short, the concepts or senses that constitute the meanings of the words they know seem to be differently organized from those of TD children. Finally, Naigles and Tek (2017) report work on young autistic adults by Perkins et al. (2006) which shows that they may use a word appropriately in a context without understanding its meaning (e.g., a young woman asked ‘what does *amplifier* mean?’, having just used it appropriately twice in a context); this may be explained by the noted strength in autism of rote-learning, alongside difficulty with flexible word use.

Summing up, Naigles and Tek (2017, p. 3) say: ‘when the appropriate comparisons are made, deriving meaning in a language context is shown to be disproportionately impaired in ASD, as is reflected in deficiencies in pragmatics and lexical semantics, whereas form or syntactic knowledge is shown to be either intact or proportional to other areas of functioning’.^22^ However, it is not clear that the kind of evidence that Naigles and colleagues present of autistic children having a compromised or atypical lexicon is a direct reflection of their well-known social-communicative/pragmatic difficulties rather than of other aspects of their conceptual cognition, in particular, their focus on specific details and differences as reflected in their impaired category induction and atypical or absent grasp of prototypes. Thus, it would be rash to claim that the work reported so far by these researchers provides direct support for the kind of pragmatically-oriented communicational lexicon I am advocating (Section 3.3). It does, nonetheless, I think, support the more general point that the formal computational side of language and the lexical meaning/conceptual side occupy different places in the overall architecture of language, as on the narrow and broad construals of language (Section 3.2), and follow different developmental trajectories.^23^

### 4.2. Homesign (language from the ground up)

The phenomenon of Homesign provides a markedly different sort of case of a special population of communicators, one which, I believe, demonstrates in a very vivid way the pragmatic/communicational nature of the lexicon. This is the case of deaf children who are as socially attuned and interactive as typical children but who, due to their circumstances (they are born into hearing families who do not know/use any conventional sign language), have no access to a public lexicon of sense conventions. In her extensive study of their communicative development, Goldin-Meadow (2003) shows how these children, who are essentially receiving no linguistic input, spontaneously employ gestures to communicate with their non-signing families, and develop a large set of signs/words, consisting of discrete gestures paired with concepts/senses. These are negotiated and calibrated in the process of intentional communicative interactions (i.e., pragmatically), functioning initially as *ad hoc* words, whose sense has to be pragmatically inferred by the interlocutor (there are inevitably failures, leading to modifications of a gesture by the child, or, in some cases, its abandonment).

As Begby (2017) puts it: ‘… homesign offers a vivid illustration of the central Relevance-Theoretic claim that ostensive-inferential processes are autonomous and can serve the ends of communication even in the absence of a conventional code’ (p. 699), and ‘individual homesign gestures are possessed of meaning, however much that meaning fails to fall under any sort of pre-existing public norm’ (p. 698), that is, these are instances of occasion-specific speaker meaning. Each of these *ad hoc* words and senses has the potential for conventionalization via frequency of use and weight of precedence, and they are not merely iconic but have a degree of arbitrariness typical of conventional word senses (Goldin-Meadow, 2003, pp. 186–87). In multiple respects, the gestures/signs produced by homesigners resemble those in established sign languages rather than the co-speech gestures of hearing people. However, it is unclear how often these do become conventionalized (and thus available for building up polysemy families) due to the exigencies of the homesigning situation: the carers’ native language and the vast majority of their language use is spoken, and, even with the best will in the world, they tend not to develop much in the way of a shared lexicon with the children (Begby, 2017, pp. 707–708). This is, therefore, not a matter of cognitive limitations of the deaf child but rather of the environment in which Homesign develops, which changes dramatically if/when the child enters into a community of peers (typically in a school), as in the well-documented case of the Nicaraguan deaf children who developed a full-blown sign language over two cohorts of schooling (Senghas et al., 2005; Brentari and Coppola, 2013).

One thing is very clear: even if polysemy is lacking or scarce in Homesign, the reason for this is very different from its absence in the autistic case which, as discussed in the previous section, seems to stem from inflexibility in word use, perhaps itself due to difficulty in grasping relevant relations between meanings. The deaf homesigners, on the other hand, are highly creative in the gestures/signs they invent and modify in their drive to communicate with their caregivers. Begby (2017) gives several examples of sign usage by the homesigning child that indicate the ability to use a single sign/gesture for more than one purpose, including the following where a child is referring to her sled ‘by a gesture indicating an imaginary wall space and a nail on that wall (indicated by hammering motion), this being the nail on which the sled usually hangs.’ (Begby, 2017, p. 707). This is quite a complex usage (what Begby calls a ‘double displacement’); simplifying somewhat, it involves the use of a homesign which means the hammering of a nail in a particular location but which is being used to refer to another object (the child’s sled); in effect, this is a ‘location for entity’ metonymy. Whether this, in fact, became an established usage for this particular small group of interlocutors (the child and family/carers) is not clear and does not much matter – it is an *ad hoc* use of a sign, which has the potential to conventionalize and so make that sign polysemous (see also Goldin-Meadow, 2003, p. 186–188).

Furthermore, the children combine these gestures into complex structures with many of the hallmarks of typical human syntax: consistent word order, predicate frames, theta roles, hierarchical phrase structure, and recursion (Goldin-Meadow, 2003, p. 97–123). Given the complete absence of linguistic input, it is hard to envisage any explanation for this other than that a syntactic system emerges (or grows) in these children on the basis of an inbuilt language faculty, just as Chomsky has maintained.^24^ While the autistic children studied by Naigles and colleagues, as discussed in the previous section, generally have good morpho-syntax, they do receive considerable linguistic input (assuming they are not deaf) and, even if they are not much interested in communication, it might reasonably be supposed that their linguistic environment is sufficient to trigger the unfolding of these formal aspects of their language. The important point for my concerns is that in both groups of children, homesigners and verbal autistic children, what we see is quite a degree of independence in the development of the formal and the meaning components of language. Linguistic form apparently came relatively easily to the autistic children but meaning was hard, leading to quite atypical lexicons, while the homesigners created meaningful words/signs as a key part of their social-cognitive drive to communicate and basic components of syntax seemed to emerge as soon as they started to combine those signs, as they do for typically developing children. While meaning (hence the communicational lexicon) is very largely dependent on social-pragmatic interaction, the organizing principles of syntax are not.^25^

## 5. Conclusion: the two parts of the language code

Decades of work within Relevance Theory has focused on the respects in which the decoded aspects of an utterance fall short of determining the speaker’s meaning, with emphasis on the extensive role played by pragmatic processes of context selection, disambiguation, reference fixing, so-called ‘free’ enrichment (contributing to the explicature) and implicature derivation. What then is the coded part of linguistic communication? The RT answer (or assumption) has been that it is a ‘semantic representation’ or ‘logical form’, which is a conceptual structure, the conceptual part coming from the lexicon (the content of substantive words being concepts), the structural part coming from the syntax. It is typically propositionally underspecified (so a conceptual schema or blueprint) and will have multiple propositional realizations dependent on the pragmatics of different contexts of use (Sperber and Wilson, 1986/1995; Carston, 2002, 2015; Wilson and Sperber, 2004; Hall, 2008, 2009; i.a.). The central theme of the current paper is that the code has two quite distinct parts: syntax (the computational engine) and lexicon (a stored/memorized set of conventionalized phonology-meaning pairings), the one a component of the narrow language faculty, the other a component of language broadly construed, a part of the conceptual-intentional mental systems.

However, when people talk of ‘the language module’ (Fodor, 1983) or the linguistic decoding module (Sperber and Wilson, 1986/1995), this distinction is seldom made explicit. Decoding or parsing (language perception) is a matter of linguistic processing (performance), and certainly the relation of the syntactic parser to the system of syntactic knowledge (competence) has received a lot of attention and hard work. Still, the syntax/lexicon distinction as conceived here raises interesting issues, which, I think, have yet to be fully addressed. First, if words are the basic units of linguistic communication and comprehension (rather than roots), then word recognition is one of two basic but quite distinct processes in language comprehension, the other being the assignment of a syntactic structure to the incoming sequence of words. The question is, then, how do these two parts of the language decoder/module work together in utterance processing/comprehension? A second quite different sort of question is what does all this mean for language evolution? Again, there is a vast quantity of work on the possible continuities and discontinuities between animal communication systems and human linguistic communication. What the current picture indicates is that it makes good analytical sense to think about the advent of recursive syntax and of words separately, with the evolution of the latter to be viewed as arising in the crucible of communication and sociality more widely, while the former was more likely a result of internal changes to the thinking capacities of the human mind/brain.

## Data availability statement

The original contributions presented in the study are included in the article/supplementary material, further inquiries can be directed to the corresponding author.

## Author contributions

The author confirms being the sole contributor of this work and has approved it for publication.

## Funding

RC was funded by the Leverhulme Trust RF-201-078. University College London covered the publication fee (Grant No. RF-2021-078).

## Conflict of interest

The author declares that the research was conducted in the absence of any commercial or financial relationships that could be construed as a potential conflict of interest.

## Publisher’s note

All claims expressed in this article are solely those of the authors and do not necessarily represent those of their affiliated organizations, or those of the publisher, the editors and the reviewers. Any product that may be evaluated in this article, or claim that may be made by its manufacturer, is not guaranteed or endorsed by the publisher.

## Footnotes

^1^I view Donnellan (1966) as the first theorist to extend the contribution of pragmatics (of the full-blown sort, i. e. geared to the recovery of a speaker’s communicative intention) to the proposition expressed by a speaker, when he rejected both a semantic and an implicature account of the referential use of definite descriptions, suggesting instead that the attributive/referential use distinction is what he called a ‘pragmatic ambiguity’ making the proposition expressed either singular (referential) or general (attributive). Since that early prescient, albeit undeveloped, remark, many more instances of pragmatic ambiguity have been mooted (Carston, 2002; Recanati, 2004).

^2^The distinction between decoding processes and inferential processes has been somewhat reconstrued (or at least relabelled) in recent years due, primarily, to revisions in the way in which ‘inference’ is understood, so that even linguistic decoding is construed as a kind of inferential process (Sperber and Wilson, 2015, and see Sperber, 2018 for informal discussion of a terminological shift from ‘ostensive-inferential’ communication to ‘ostensive-interpretive’ communication). However, the distinction between the two kinds of process involved in utterance interpretation remains untouched and, for the time being at least, I see no harm in talking of ‘the code’, and of ‘encoding’ and ‘decoding’, taking these terms to concern the role(s) of the language system in linguistic communication, although I will suggest below that this code is a kind of hybrid, comprising two quite architecturally distinct parts.

^3^There are many more detailed discussions and exemplifications of *ad hoc* concept construction in the relevance-theoretic literature (Sperber and Wilson, 1998, 2008; Carston, 2002,^ 2019^, ^ 2021^; Wilson and Sperber, 2002, 2004; Wilson and Carston, 2007; Falkum, 2017).

^4^For another, quite different, application of *ad hoc* concepts within the philosophy of language, see Liu (2023), who argues that phenomenal ‘what-it’s-like’ concepts typically originate as *ad hoc* concepts (pragmatically narrowed) and that this presents a challenge for work in experimental philosophy that tests whether laypeople grasp these concepts and draws conclusions from their apparent failure to do so.

^5^I should point out that the ideas of Wilson and Falkum that I draw on here come from a series of conference presentations, and may not represent their final position when the account is published. In her work on referential metonymy, Bowerman (2019, 2021) takes a somewhat different but possibly compatible position, according to which a metonymic use of a word or a phrase (e.g., ‘the ham sandwich’) is a *repurposing* of the literal meaning of the expression in order to facilitate the interpreter’s access to a novel referent (e.g., a customer who ordered a ham sandwich), on the basis of a contextually salient relation between its literal referent (e.g., an actual ham sandwich) and the speaker’s target.

^6^As noted by Bauer (2018), others have also suggested that conversions are cases of metonymy or discussed the pros and cons of the idea (e.g., Dirven, 1999; Cetnarowska, n.d.).

^7^One of the reviewers raised the interesting question whether new words (derived from phonologically identical ones) must always involve a metonymic relation between senses and suggested that the relation might sometimes be metaphorical. They further suggested that the verb ‘houdini’ might be such a case, being based on a resemblance between our concept of the man Houdini and our concept of the action of escaping in an incredible way. While I am doubtful about this specific example (it does not seem that the individual concept encoded by the name ‘Houdini’ is modulated into a verbal (action) concept meaning ‘to escape incredibly’), I would agree that the senses of some new words may bear a metaphorical relation to their origin word’s sense – this is something that definitely needs further consideration. For now, I follow Wilson & Falkum’s (unpublished) work on this and take the following conditional position: if the pragmatic relation between a new/*ad hoc* sense and an existing sense of a single phonological form is metonymic, then the new sense is the meaning of a new word (or new communication unit).

^8^It has been suggested by Dan Sperber (2017) that non-human primates may also use metonymy. Here he draws on experimental work with apes by Bohn et al. (2015), in which chimps, bonobos and baboons pointed through a wire mesh to an empty plate in order to request a certain preferred kind of food (grapes) which they had previously received on that plate, which looks very much like a case of *container for contents* metonymy. This may be an evolutionary precursor to the human use of metonymy, but it is rather different from human metonymy because only humans have words, that is, phonological units with syntactic structure which couple up with (families of) discrete atomic concepts/senses (Pettito, 2005).

^9^I say ‘so-called’ conversions because on the constructionist account (Borer, 2013a, 2014), there is no ‘conversion’ process: e.g. the noun and verb pair ‘hammer’ simply arise from insertion of the root √hammer into two distinct formal structures, each of which is a domain for atomic meaning/Content. It is completely irrelevant to the syntactic system which of these, noun or verb, ‘came first’, in terms of its coinage and use by communicators and its storage in their lexicons.

^10^Another very significant class of words, namely, compounds (e.g., ‘skyscraper’, ‘flowerbed’, ‘eavesdrop’, ‘earmark’) needs to be discussed in this regard. They are another case whose meaning seems clearly non-compositional (most strikingly so in the case of ‘eavesdrop’), hence requiring a pragmatic account (see Bezuidenhout (2019) on the semantics/pragmatics of noun-noun compounds in English). In her study of noun-noun constructs in Hebrew, Borer (2013b) shows that, from the point of view of (non)-compositionality, there are two kinds of case such that, as with the nominals discussed above, ‘it is the syntactic differences between them that give rise to distinct Content properties, with non-compositionality correlating with … the absence of functional structure’ (Borer, 2013b, p. 205).

^11^See Durrleman et al. (2022) for some intriguing empirical evidence from bilingual children with atypical development which they interpret as supporting the chomskyan position that: ‘language, though useful for communication, is not sufficient for communication, and may arguably not have evolved primarily for communicative purposes [but for thought]’ (Durrleman et al., 2022, p. 5).

^12^As Markson and Bloom (1997, p. 815) note: ‘Children are much better at learning phonology, morphology and syntax than adults, consistent with the notion of a biological specialization for these aspects of language’. That is, there appears to be a critical period for acquisition of the structural components of language, but not for the acquisition of words.

^13^This is not to say, however, that children do not appreciate a difference between words for things and facts about things (‘things’ used broadly here to cover not just entities, but also processes, activities and events in the world); they certainly do, especially concerning the conventionality of word meanings, and key differences between kinds of facts: generalizations (‘Dogs bark’) and one-off facts (‘Uncle John gave me this’), only the former playing a useful role in categorization (as do words). See Tippenhauer and Saylor (2019) for a balanced overview of the ways in which words and facts, and their learning, overlap and differ.

^14^Julien (2007), who argues strongly against words having any scientific or theoretical reality, nevertheless recognizes their psychological reality to us as language users, which she says is probably due to their distributional properties: ‘since words are the minimal morpheme strings that can be used as utterances and that may be permuted more or less freely, words are the minimal linguistic units that speakers can manipulate consciously. It is therefore no surprise that speakers are generally aware of words’ (Julien, 2007, p. 83).

^15^Another important property of our lexicons is discussed by Clark (1998), which is that it includes various ‘communal sublexicons’, that is, we index or tag conventionalized senses of words to the specific community for whom we take them to be conventional (economists, linguists, Londoners, football aficionados, etc.) and deploy them accordingly.

^16^Another ‘pragmatics first’ approach is that of Christiansen and Chater (2022), who show how the gradual conventionalization of gestures/signals in the game of charades can capture, in miniature, some crucial aspects of the cultural evolution of language, specifically words. As with the epidemiological approach, this seems promising as an account of the establishment of words and senses (basic units of human communication), but less so for the much more rigid structures of syntax that speakers seldom innovate with.

^17^Based on work in psycholinguistics on production (e.g., picture-naming tasks), Ramchand (2022) suggests that lexical entries are ‘hubs’ or ‘lemmas’, in the sense of Levelt (1999), that is, they house all inflectional forms of the ‘same’ lexeme (e.g., singular and plural for nouns; tense/aspect for verbs), while distinct derivational items based on a single stem are distinct words with distinct lexical hubs (e.g., ‘form’, ‘formation’, ‘formative’).

^18^Alerted to current guidelines by one of the reviewers, I have changed my earlier use of the expression ‘children with autistic spectrum disorder (ASD)’ to ‘autistic children’ (except when quoting others). However, it is clear from the literature on autism that the people to whom the adjective ‘autistic’ is applied vary considerably in the degree to which they manifest associated abilities and disabilities, so the word ‘spectrum’ seems appropriate and helpful.

^19^They suggest that the absence of the polysemy advantage that TD children exhibit may be due to the well-known general tendency of autistic people to focus on ‘specifics rather than on relationships among entities’.

^20^Also highly relevant to the autistic children’s difficulty in allowing words to have multiple related meanings and so to become polysemous is their widely reported literalism, that is, their tendency to give literal interpretations to language intended non-literally (e.g., metaphorically or metonymically). See Vicente and Falkum (2023) for a review and critique of explanations of this tendency, and exposition of their own view that literalism is a result of the more general autistic characteristic of strong adherence to rules and conventions.

^21^One of the reviewers of the paper maintains that the evidence concerning autistic people’s formal syntactic abilities is far from consistent. They note one study (of a small group of autistic adults) in which the situation seems almost the opposite to that of the autistic children surveyed by Naigles and Tek (2017), in that the participants (all of whom had very low receptive vocabularies), were unable to comprehend NP’s composed of nouns and adjectives that they understood in isolation (Vicente et al., 2023). Certainly, a lot more work is needed before any general conclusions can be drawn about the formal linguistic abilities of the wide spectrum of people diagnosed as autistic.

^22^Directly parallel results have been found in recent work testing pre-school Chinese autistic children (Su and Naigles, 2022).

^23^Many thanks to Agustin Vicente for valuable help with this section, especially his detailed cautionary remarks about what implications the work reported here could (and could not) have for my position.

^24^See also Carrigan and Coppola (2012), who show that even when caregivers do their best to communicate with the child in Homesign, the gesture combinations they produce lack the morphological and syntactic structure that is observed in the child’s productions, so it is not the caregivers’ input that is driving the child’s formal linguistic development.

^25^It follows then that, while the view that all language acquisition is a product of language use in communication (the ‘constructionist’ position held by Tomasello (2008) and many others) seems wrong for syntax, it may well be the right way to think about the development of a communicational lexicon. See also Kissine (2021), who argues that the patterns of language acquisition and learning in autism present a strong challenge to constructionist theories of language development.
